# Large Group Exposure Treatment: A Feasibility Study of Exposure Combined with Diaphragmatic Breathing in Highly Dental Fearful Individuals

**DOI:** 10.3389/fpsyg.2016.02007

**Published:** 2017-01-06

**Authors:** André Wannemueller, Hans-Peter Jöhren, Alina Borgstädt, Jessica Bosch, Milena Meyers, Miriam Völse, Saskia Scholten, Jürgen Margraf

**Affiliations:** ^1^Department of Clinical Psychology and Psychotherapy, Mental Health Research and Treatment Center, Ruhr-University BochumBochum, Germany; ^2^Dental Clinic BochumBochum, Germany

**Keywords:** exposure, exposure treatment, group treatment, diaphragmatic breathing, one-session treatment, dental fear, dental phobia

## Abstract

A large-group one session treatment (LG-OST) combining exposure and diaphragmatic breathing as a bodily coping element was carried out to investigate its feasibility and effectiveness in a sample of 43 highly dental fearful individuals treated simultaneously. We assessed subjective dental fear, dysfunctional dental-related beliefs, and perceived control pre- and post-intervention and at four-month follow-up. Participants additionally performed a behavioural approach test (BAT) pre- and post-intervention. During the applied exposure exercises, four participants (9.3%) discontinued the program all reporting too high levels of distress. Regarding subjective dental fear and dysfunctional dental related beliefs post treatment effects, LG-OST showed medium to large effect sizes, ranging from Cohen’s *d* = 0.51 to *d* = 0.84 in the Intention-to-Treat analysis. Subjective dental fear improved clinically significantly in about one fourth (25.6%) of therapy completers. All post-treatment effects remained stable over time. Concerning the behavioral fear dimension, we observed a strong ceiling effect. Already at pre-assessment, participants accomplished more than six out of seven BAT-steps. Thus, behavioral approach did not increase significantly following treatment. Overall, the LG-OST protocol proved feasible and efficient. Compared to other one-session individual and multi-session group treatments the observed LG-OST effects were smaller. However, if LG-OST could match the efficacy of highly intensive short treatments delivered in an individual setting in the future, for example, by applying a wider array of exposure exercises, it could be a very useful treatment option as an intermediate step within a stepped care approach.

## Introduction

Specific Phobias (SP) are proven to be of high health-economic relevance, although they are widely considered to be less restrictive than other mental disorders. With a 12-month prevalence rate between seven and nine percent in Western countries (American Psychiatric Association, 2013), about 22.7 million people in the European Union suffered from a specific phobia in 2010 (Wittchen et al., 2011). SPs are the most common anxiety disorder and following major depression, they are the second most prevalent of all psychiatric disorders (Wittchen et al., 2011). The impairments caused by SPs are widely underestimated. A survey conducted by the German Federal Ministry of Health (Wittchen and Jacobi, 2004) reported the risk of being absent from work to be twice as high in women who suffer from SP and even fourfold in phobic men.

Dental Phobia (DP) is among the most critical SPs in terms of both prevalence and impairment (Oosterink et al., 2009). About 20% of adults claim to be highly anxious when thinking about upcoming dental surgery (Oosterink et al., 2009) and 5% (Enkling et al., 2006) avoid dental treatments altogether. Point prevalence of DP ranges from 2.1% (Frederikson et al., 1996) to 3.7% (Oosterink et al., 2009). In individuals suffering from DP, serious dental health indices have been reported with about eight teeth currently requiring dental treatment (Thom et al., 2000; Wannemüller et al., 2011). The risk of somatic comorbidities, such as cardiac disease (Cronin, 2009) resulting from poor dental health, as well as psychiatric comorbidities such as other anxieties, mood disorders or substance abuse (Roy-Byrne et al., 1994) is greatly enhanced in individuals suffering from DP.

Considering the sheer number of afflicted individuals and limited number of clinical professionals, the existing health care system cannot provide sufficient treatment options for all. Therefore, more efficient treatment approaches that are short in time and minimize the patient to therapist ratio would represent a very useful treatment option; especially in those mental disorders that are comparably moderate and for which well-established treatment forms already exist, as both is the case in SP.

Exposure-based treatments have been proven to be highly effective treatment tools in SP. The long-term effects following treatments provided in multi-session formats were shown to slightly outperform the effects of treatments providing exposure in one-session. However, such one-session treatments (OST) have been demonstrated to reduce SP-symptoms very effectively (for a review see Wolitzky-Taylor et al., 2008). Originally, OSTs lasting for 3 h or less, first introduced by Öst (1989), consisted of exposure *in vivo* and elements of participant modeling. However, cognitive and motivational aspects have been added through psychoeducative elements, skills training, reinforcement, and cognitive challenges (see Zlomke and Davis, 2008 for a review). During a typical OST, the therapist encourages the patient to interact with the feared stimulus by mastering a step of a subjective fear hierarchy. The therapist challenges patient’s beliefs in the context of the fear-evoking stimulus and motivates him to emulate. Later on, the therapist’s support is restricted to instructions and presence only. So far, OSTs have been successfully applied in a wide range of phobic disorders: spider phobia (i.e., Öst et al., 1991; Hellström and Öst, 1995), flight phobia (Öst et al., 1997), injection phobia (Öst et al., 1992), and agoraphobia (Öst et al., 2001). Across phobic disorders, OSTs show high efficacy in adults with clinical improvement rates of 80–90% (Zlomke and Davis, 2008). However, the efficiency of OSTs is limited, as long as they are conducted in individual settings.

As in other SP, in DP (Haukebø et al., 2008) and intra-oral injection phobia treatment (Vika et al., 2009), OSTs comprising direct and modeled exposure elements have been proven to be effective. An OST-format exclusively consisting of cognitive restructuring (De Jongh et al., 1995a) also led to a substantial loss of subjective dental fear long-term. As already mentioned, with regard to dental fear, effectiveness of multi-session group treatments delivered in small groups has repeatedly been reported (Ning and Liddell, 1991; Liddell et al., 1994; Moore et al., 2002). Similarly, a recent study reviewing the effects of dental fear treatments (Gordon et al., 2013) concluded that short CBT-interventions up to five sessions were effective both short-term as well as at follow-up (FU) assessment, regardless of their respective content elements (i.e., exposure with relaxation, cognitive restructuring, cognitive and behavioral approaches combined), format (e.g., individual or group), intensity (e.g., massed, graduated), or frequency (e.g., one session, five consecutive sessions). Efficacy of group treatments that reduce the therapist-patient ratio has been repeatedly demonstrated in SPs, for example, in acrophobia (Ritter, 1969; Pendleton and Higgins, 1983), flying phobia (Howard et al., 1983), or DP (e.g., Ning and Liddell, 1991; Moore et al., 2002). However, treatment in these trials was applied in a multi-session format and the group size was small with at the most nine participants.

So far, there have been only a few attempts to combine the advantages of OSTs and the group setting for treatment efficiency, i.e., a short treatment duration and favorable patient to therapist ratio. Three studies delivered OSTs in a small-group format consisting of groups up to eight participants (Öst, 1996; Öst et al., 1997; Götestam, 2002). They all targeted spider fear and were based on modeled exposure strategies. All studies reported good treatment efficiency. Moreover, Öst et al. (1991) demonstrated that in most measures the effects of smaller groups (*n* = 3–4) did not significantly differ from effects in larger group conditions (*n* = 7–8). With the aim to significantly increase treatment efficiency, a recent study (Wannemüller et al., 2016) applied indirect participant modeling strategies in a large-group OST-setting consisting of 78 highly spider-fearful individuals treated simultaneously. The authors demonstrated feasibility of the procedure, which led to substantial fear reduction at post treatment and at FU-measurement. However, participants treated with an individual OST performed better, especially in regard to behavioral fear reduction. The authors concluded that more research on large-group treatments with individuals suffering from more restricting fears is needed to evaluate the potential value of large-group treatments.

Taken together, there is good evidence for the effectiveness of one-session as well as of group treatments in SP, including DP. However, so far only a few attempts have combined the advantages of OSTs and the group setting with promising results in terms of treatment efficiency. All except for one were conducted in small groups. A large-group OST trial conducted in spider fear proved feasible and effective. The current study focused on the evaluation of feasibility of large-group one session treatment (LG-OST) in a sample of highly dental-fearful individuals.

The present study aimed to investigate whether a LG-OST containing well evaluated exposure and coping strategies is feasible and effective in a sample of highly dental-fearful individuals. LG-OST evaluation contained subjective, cognitive and behavioral fear dimensions as well as dental-related control in order to investigate treatment efficacy on all levels afflicted by clinical dental fear as well as subjective therapy success. Following the concept of evidence-based psychotherapy (Norcross et al., 2005), the design of this Phase I open trial did not contain a direct control group condition. We also aimed to investigate whether inter-individual differences in trait anxiety or depressiveness are associated with LG-OST benefits. Such results could provide first indications of the potential target group for LG-OST as a useful treatment option.

## Materials and Methods

### Participants

For this Phase I investigation of LG-OST 54 individuals registered online on a website established for this project. Most of them^1^ were recruited via ‘Dental Clinic Bochum’; a dental clinic specialized in treating dental fearful individuals. There, during initial anamneses, dental fear is routinely screened using the ‘Hierarchischer Angstfragebogen’ [Engl. transl. ‘Hierarchical Fear Questionnaire,’ HAF, Jöhren (1999), for a psychometric description of the HAF see the questionnaire section]. Individuals who came to the dental clinic between August 2014 and January 2015, exhibited a heightened fear score in the HAF (>35) and reported that they had avoided dental surgeries due to dental-fear for more than two years were informed about LG-OST and encouraged by the dentists to register for the LG-OST-program online. Besides subjective high fear, being full-aged (18 y) was required for participation. Finally, 43 participants, all Caucasian (34 female) with a mean age of 50.56 (*SD* = 11.30) years attended LG-OST. Eleven individuals who preliminarily registered failed to attend. Thirty-nine of the 43 participants completed the LG-OST-program. Four participants (9.3%) dropped out during the training. They discontinued the intervention during video exposure and reported that their stress-levels were too high as the reason for premature termination.

**FIGURE 1:**
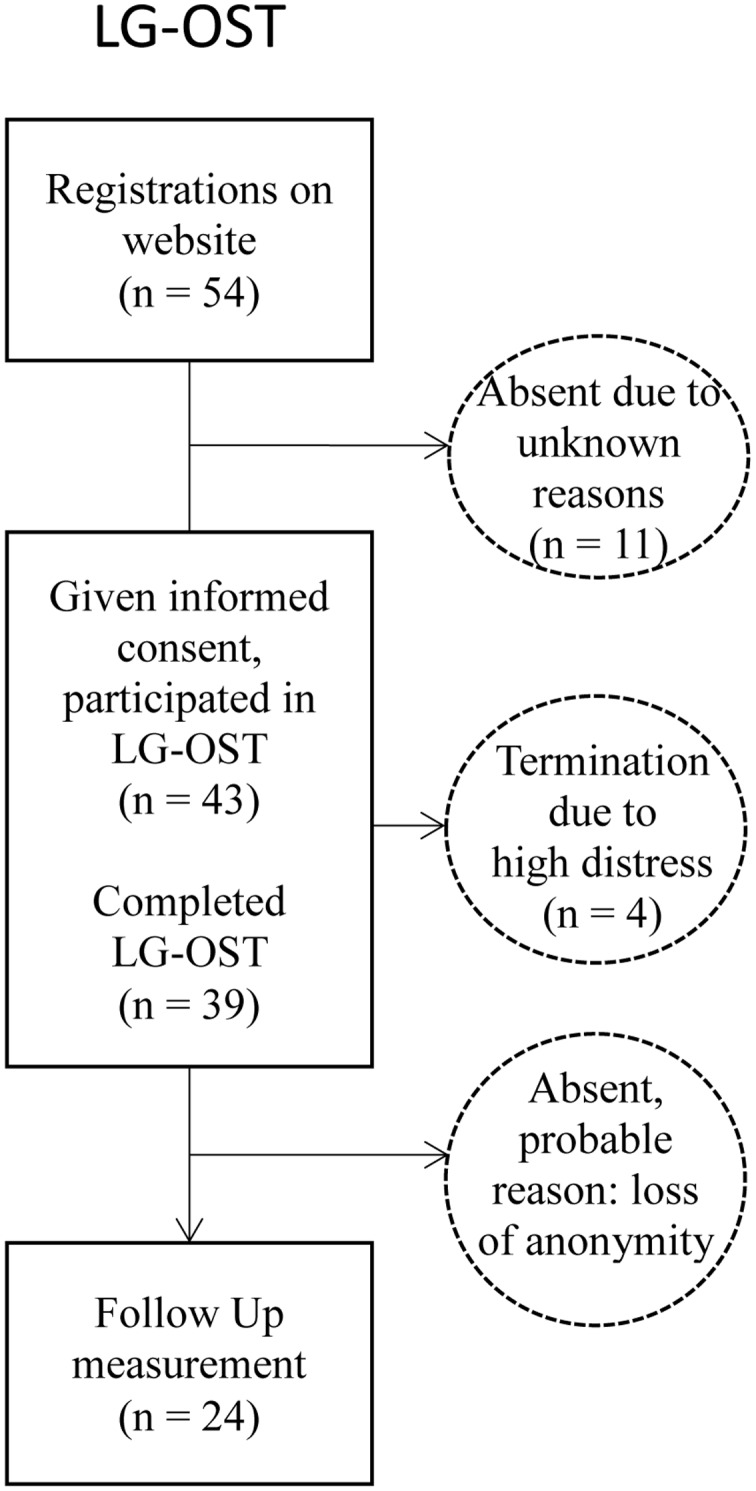
**Flow diagram of patients entering the study**.

The LG-OST trial was not preceded by a diagnostic session. Hence, symptom information of LG-OST participants is solely based on questionnaire data (**Figure 1**).

### Procedure

The LG-OST-website informed about anonymity during treatment. Therefore, we aimed to reduce the subjective threshold to participate in the large-group format. LG-OST was preceded and followed immediately by a behavioral approach test (BAT). During the BAT, participants went through several steps that typically occur during dental treatment. Before leaving, all participants were asked to voluntarily sign up and to be available for FU-measures. Twenty-four participants (55.8%) signed up for FU. Moreover, participants completed a set of questionnaires, assessing subjective, and cognitive components of dental fear pre- and post-intervention. After 4 months, we invited the participants for FU-measurement via e-mail and provided access to a secured website. There, they completed the same questionnaires as at pre- and post-LG-OST online. The mean post-FU interval was 4.31 (*SD* = 0.57) months.

The LG-OST was carried out by a postgraduate clinical psychologist, well-trained in treating specific phobia, and specialized in DP treatment. The local Ethics Committee of the psychology faculty of the Ruhr-Universität Bochum, where the study was conducted, approved the study. Informed consent procedure was carried out with participants.

### Treatment

The treatment was applied in one session with a total duration of 140 min in an auditorium at the Dental Clinic Bochum. Treatment consisted of three phases:

#### Psychoeducation (ca. 40 min)

Initially a video was presented showing a dentist specialized in the treatment of dental fearful patients. He dealt with common myths about dental surgery (i.e., probability of pain, lack of control, etc.). Afterward, a psychotherapist explained the nature and utility of fear and its cognitive, behavioral and subjective consequences. He outlined the aim of the following treatment as a strategy to cope with fear responses emerging during the following exposure exercises.

#### Diaphragmatic Breathing (ca. 20 min)

Instructed by the psychotherapist, participants learned a form of diaphragmatic breathing via an active exercise. They were told that relaxed breathing could markedly decrease sympathetic activity and reduce stress and tension (Busch et al., 2012). The therapist encouraged participants to apply the deep breathing technique during the following exposure exercises in order to deal with upcoming fear responses.

#### Exposure (ca. 80 min)

Exposure to fear-related contents was delivered via a video professionally produced for the project. In this video, a complete dental surgery (construction filling of a carious tooth) was shown in real time from the patient-perspective. The video starts at the point when the patient enters the doctor’s office and ends when he leaves. Afterward, we conducted in sensu exposure containing tooth-filling as a stimulus-set. During this exercise, the therapist encouraged patients’ imaginal exposure by providing a detailed description of every step of surgery and triggered subjects’ fear by stating some common physiological and cognitive fear symptoms (racing heart, tensed body, feeling helpless, thinking about canceling the surgery). He instructed the participants to counteract physiological fear responses by applying the breathing technique. At the end of exposure, the therapist encouraged the patients to undergo dental surgery in the near future and to overcome dental fear in a real life situation. Presented in an individual setting, the program yielded very high effects on subjective and behavioral fear responses in a pilot-study (Wannemüller et al., 2015). A three-session individual version of the treatment evidenced significant predominant effects compared to two forms of dental-hypnosis (Wannemüller et al., 2011).

### Measures

#### Behavioral Approach Test (BAT)

During the BAT, participants were asked to accomplish seven steps: (1) enter a dental treatment room; (2) take a seat in the dental chair; (3) permit the dentist to move the chair into a horizontal position; (4) allow the dentist to move the tablet with dental instruments over the body; (5) open the mouth and permit the dentist to look into the mouth using the speculum; (6) permit the dentist to remove tartar with a dental probe; (7) allow the dentist to switch on a dental drill and enter the opened mouth. Before each step, the intended activity was clearly announced and participants had to explicitly give their consent to proceed. A step was scored as completed if the person could endure the situation for at least 10 s.

#### Subjective Dental Fear

The German translation of the *Dental Anxiety Scale* (*DAS*; Corah, 1969) was used to assess anticipatory and subjective dental fear in four hierarchical situations on a 5-step Likert-scale (1 = relaxed; 5 = to be feared sick). Internal consistency (Cronbach’s α) of the German DAS was reported to be α = 0.64 (Sartory et al., 2006). We found a score of α = 0.82 in our sample. The German ‘*Hierarchischer Angstfragebogen*’ (HAF; Jöhren, 1999) consists of 11 items measuring subjective dental fear. Patients rate how much anxiety they would experience in 11 hierarchically ordered phobic situations on a scale of 1–5. The cut-off score for DP is 38. An internal consistency index of Cronbach’s α = 0.80 has been reported by the authors. We found an internal consistency of Cronbach’s α = 0.92 in the present sample.

#### Dysfunctional Dental-Related Cognitions

Dysfunctional dental-related cognitions were assessed with the German translation of the *Dental Cognition Questionnaire* (*DCQ*; De Jongh et al., 1995b, German Version). The DCQ consists of 38 dichotomous items (yes/no) measuring dysfunctional dental-related cognitions that might emerge prior to (Items 1–14) or during dental surgery (Items 15–38). The sum of yes-answers constitutes the ‘frequency’ scale of the DCQ. Furthermore, the DCQ offers a ‘believability’ score consisting of the mean percentage of approval (not agree – total agree) for each item answered with ‘yes.’ The authors of the DCQ report a high internal consistency of the frequency-scale (Cronbach’s α = 0.89). However, due to its high error-susceptibility and low consistency we did not include the DCQ- ‘believability’-scale in our analyses.

#### Dental-Related Control

The *Iowa Dental Control Index Revised* (*IDCI-R*; Brunsman et al., 2003, German version) consists of nine items measuring ‘predicted control’ (four items) and ‘desired control’ (five items) during dental surgery. Items were answered on a 5-point Likert-scale (0 = no control at all; 5 = total control). Individuals showing high discrepancy between predicted and desired control expressed highest distress and suffering from dental treatments (Logan et al., 1991). Acceptable internal consistencies were found for the English language IDCI-R with Cronbach alphas of 0.82 and 0.74 for ‘desired control’ and ‘predicted control,’ respectively. In our sample, Cronbach’s α was 0.82 (‘desired control’) and 0.74 (‘predicted control’).

#### General Clinical State

A German 21-item version of the *Depression Anxiety Stress Scale* (DASS; Lovibond and Lovibond, 1995) was used. This self-rating questionnaire measuring negative emotional status is answered on a 4-point scale (0 = never – 3 = almost always). Besides an overall score including all 21 items, the DASS provides three separate scales, each consisting of a seven-item set: depression, anxiety and stress. In our analyses we only used the overall score which in our sample had an internal consistency of Cronbach’s α = 0.92.

The German version of the *State-Trait Anxiety Inventory* (STAI; Laux et al., 1981) consists of two subscales, each describing an emotional state in 20 statements at present (state) and in general (trait version). Scores range from 20 (no anxiety) to 80 (high anxiety). Whereas the state-scale is highly sensitive for change, the trait-scale has a high retest reliability (*r*_tt_ < 0.96).

#### Global-Success-Rating (GSR)

On a 7-point Likert-scale, subjective state changes from 1 (much worse) to 7 (much better) are rated. A score of 4 indicates no subjective change.

### Statistical Analysis

Because dental related measures were likely to be correlated, we conducted a 2 (time) x 6 (measure) MANOVA with repeated measures containing all questionnaire measures associated with dental fear (DAS, HAF, DCQ, IDCI-R desired, IDCI-R predicted) and the BAT to analyze pre to post changes. It was carried out as an intention-to-treat analysis (ITT). Within the ITTs, the last observation carried forward (LOCF) method was used to impute missing data at post assessment. FU-analyses were carried out as completer-analysis, again using repeated measure MANOVAs. Within-group effect sizes were calculated using Cohen’s *d* formula based on pooled standard deviations (Cohen, 1988). Moreover, $\eta_{p}^{2}$ values as measures for effect-size were given for within- and between-group comparisons.

Furthermore, we conducted analyses on ‘clinically significant improvement’ in therapy completers. In default of a non-clinical control sample, we defined ‘clinically significant improvement’ according to Jacobson et al. (1984) criteria. Following this, a patient should, besides showing a statistically reliable change to post-treatment (we defined this as at least a 20% symptom change from pre to post) lie outside the range of the clinical sample, that is, mean ± 2 SDs. Applying this formula, we calculated limit values for the DCQ (i.e., DCQ post-score ≤ 9) and IDCI-R p (i.e., IDCI-R ≥ 14). In case of the DAS, we applied a score of DAS < 13, since this is a common cut-off score for high dental fear and phobia (Corah et al., 1978). Because we observed a strong ceiling effect in the pretreatment BAT, we did not include the BAT in our analyses on clinically significant improvement.

To identify variables that were significantly associated with subjective dental fear decrease following LG-OST, we conducted Pearson-correlation analyses between the change score of the DAS from pre to post and age, clinical baseline-status (DASS, STAI-Trait) and all dental fear pre scores. All analyses were conducted using the IBM SPSS 23 Statistics program.

## Results

### Is LG-OST Effective in Reducing Dental Fear at Post Treatment?

MANOVA showed a highly significant main effect of time [*F*(6,29) = 8.41, *p* < 0.001, η^2^ = 0.64]. Associated univariate tests showed highly significant treatment effects in regard to both measures assessing subjective dental fear [DAS: *F*(1,34) = 37.87, *p* < 0.001 η^2^ = 0.53; HAF: *F*(1,34) = 16.67, *p* < 0.001 η^2^ = 0.33]. The same was true for dysfunctional beliefs [DCQ *F*(1,34) = 29.41, *p* < 0.001 η^2^ = 0.46]. Furthermore they showed that participants’ predictions in regard to control (IDCI-R p) during dental surgery [*F*(1,34) = 6.71, *p* = 0.014; η^2^ = 0.17) and approach behavior (BAT) increased significantly [*F*(1,34) = 5.07, *p* = 0.031; η^2^ = 0.13]. However, some participants did not complete the DCQ correctly at pre assessment, which is why we only could apply data of 35 participants in the MANOVA. Therefore, in **Table 1** we report the results of single ANOVAs with repeated measures conducted for each measure separately. In terms of *p*-levels there was only one difference between the MANOVA’s and the single ANOVAs’ results: the BAT changes following treatment did no longer reach the level of significance. In this context, it is worth noting that already at pre-assessment, participants accomplished more than six out of seven BAT-steps (see **Table 1**).

**Table 1:** Sample characteristics, ITT-means, (SDs) and effect strengths (Cohen’s *d*) of pre to post changes of dental fear measures within the LG-OST-condition.

	LG-OST (*n* = 43)			
	
	Pre	Post	Pre > Post	
			
	*M (SD)*	*M (SD)*	*statistics*	*ES^a^ [95% CI]*
*Sample characteristics and clinical data*
Sex (f/m)	34/9			
Age (years)	50.56 (11.30)	–	–	–
STAI-S	46.78 (11.67)	43.16 (12.77)	*F*(1,42) = 6.17^∗^, η^2^ = 0.13	0.30 [(-0.13)–(0.72)]
STAI-T	43.83 (11.44)	–	–	–
DASS	19.98 (12.32)	–	–	–
*Dental fear measures*			
DAS	16.95 (2.49)	14.44 (3.40)	*F*(1,42) = 44.37^∗∗∗^, η^2^ = 0.51	0.84 [0.40–1.29]
HAF	43.75 (6.87)	40.00 (7.75)	*F*(1,42) = 20.94^∗∗∗^, η^2^ = 0.33	0.51 [0.08–0.94]
DCQ	21.00 (5.92)	15.96 (6.33)	*F*(1,34) = 29.41^∗∗∗^, η^2^ = 0.47	0.82 [0.37–1.26]
IDCI-R p	7.95 (3.06)	9.02 (3.10)	*F*(1,42) = 6.65^∗^, η^2^ = 0.14	-0.35 [(0.08)–(-0.77)]
IDCI-R d	21.91 (3.69)	21.51 (3.69)	*F*(1,42) = 2.51, n.s.	–
BAT	6.19 (1.45)	6.35 (1.48)	*F*(1,42) = 1.60, n.s.	–
GSR	–	4.92 (0.88)	–	–


*DAS = Dental Anxiety Scale; DCQ = Dental Cognition Questionnaire; IDCI-R p = Revised Iowa Dental Control Index, predicted control; IDCI-R d = Revised Iowa Dental Control Index, desired control; GSR = Global Success Rating. ^a^Effect sizes not reported when pre- to post-changes were not significant. ^∗^*p* < 0.05; ^∗∗^*p* < 0.01; ^∗∗∗^*p* < 0.001.*

### Which Variables Are Associated with Subjective Fear Reduction Following LG-OST?

There was only one significant correlation: The lower the state-fear (STAI-State) at pre-treatment assessment, the higher the benefit in regard to subjective fear reduction (*r* = -0.32, *p* = 0.036).

### What Is the Clinically Significant Improvement Resulting from LG-OST?

Considering therapy completers, 25.6% of LG-OST participants showed clinically significant improvement of subjective dental fear (DAS) post-treatment. Clinically significant change in terms of dysfunctional dental-related beliefs (DCQ) was reported in 19.4% and regarding the perceived control dimension in 2.6% of LG-OST participants.

### Are the LG-OST Effects Stable Over Time?

Twenty-four LG-OST-participants (55.8%) were available for FU-measures. To investigate selective dropout-effects from post to FU within the LG-OST-condition, we initially compared gender, age and all pre to post outcomes for completers and non-completers with univariate ANOVAs. There were no significant differences at baseline-assessment concerning socio-demographic, clinical (DASS), or dental-fear measures between FU-completers and non-completers. The same was the case for all pre to post changes in the questionnaires as well as in the BAT (all *p* > 0.05). Hence, dropout was non-selective.

To analyze the course of dental related symptoms within the post-FU interval, we conducted a MANOVA with repeated measures within LG-OST as also applied for the pre to post analyses (for pre, post, and FU means and SDs see Table 2). The analysis did not yield a main effect of time [*F*(4,11) = 2.40, *p* = n.s.] and the associated univariate tests did not yield significant results. Single *post hoc* ANOVAs regarding the DAS, [*F*(1,22) = 0.12, *p* = n.s.] and dysfunctional dental related thoughts [DCQ: *F*(1,15) = 0.02, *p* = n.s.], did not show significant changes from post to FU. The same was the case for predicted control (IDCI-R p) during dental surgery [*F*(1,21) = 1.18, *p* = n.s.]. However, the desire for control during dental surgery (IDCI-R d), which did not change significantly from pre to post, decreased significantly from post to FU [*F*(1,23) = 4.44, *p* = 0.05, η^2^ = 0.16]. The ratings of therapy success from post to FU did not decrease significantly [*F*(1,22) = 2.78, *p* = n.s.].

**Table 2:** Means (SDs) of dental-fear measures (pre, post, FU) for LG-OST participants that completed FU-assessment.

	FU completers (*n* = 24)		
		
	Pre	Post	FU
	*M (SD)*	*M (SD)*	*M (SD)*
DAS	17.30 (2.34)	14.17 (3.55)	14.00 (3.92)
DCQ	20.77 (6.41)	15.77 (7.13)	15.00 (8.82)	
IDCI-R p	7.95 (3.00)	10.14 (2.90)	8.64 (2.59)
IDCI-R d	21.87 (3.77)	21.52 (3.60)	19.96 (3.91)
GSR	–	5.00 (0.95)	4.70 (0.82)


*DAS, Dental Anxiety Scale; DCQ, Dental Cognition Questionnaire; R-IDCI p, Revised Iowa Dental Control Index, predicted control; IDCI-R d, Revised Iowa Dental Control Index, desired control; GSR, Global Success Rating.*

### What Is the Long-Term Outcome of LG-OST Completers?

The long-term reduction of subjective dental fear in the DAS was 19.08% (change from pre to FU). Dental-related dysfunctional beliefs decreased by 27.78% from pre to FU. Regarding control (R-IDCI), we found only a small increase of 8.68% in the predicted control and decrease of 4.50% in the desired control dimension.

## Discussion

In this Phase I study, we investigated whether a OST approach combining coping with exposure is feasible and efficacious in a large group setting (LG-OST). To the best of our knowledge, dental fear treatment has not been delivered in an OST-group format so far. In terms of mere feasibility, the results were promising: participant inflow was good and the participants did not address us with concerns regarding the effectiveness of the treatment. Furthermore, we did not notice any signs of individual or even mass panic symptoms amongst participants during the large-group exposure exercises.

In fact, we observed a marked reduction in subjective dental fear, dysfunctional beliefs and loss of dental-related control from pre to post in our LG-OST group. In about one third of the participants, subjective dental fear improved clinically significantly. Four participants discontinued the treatment abruptly during video exposure. However, the total number of patients, who received the intended dose of treatment, was considerably high compared to dropout rates of up to 45% reported for other exposure based dental fear treatments (for an overview see Choy et al., 2007). This might represent a major advantage of one-session compared to multi-session treatments. We could not observe significant improvements following LG-OST in regard to the behavioral fear dimension in the BAT. However, we observed a huge ceiling effect in the BAT, with participants managed more than 6 of 7 steps already at pre-BAT. Our informal observations yielded that many of the participants showed heavy fear responses during pre-BAT and appeared much more relaxed at post-BAT. In our previous LG-OST study targeting spider fear (Wannemüller et al., 2016), we also observed a remarkably high performance at pre-BAT in our large-group participants. In that study, LG-OST participants clearly performed better in a pre-BAT compared to participants who were treated individually, although both groups did not differ in terms of their subjective spider fear levels at pre-treatment assessment. This is noteworthy since it suggests that the large-group context might have stimulated the participants to approach the feared stimulus and overcome their avoidance. Social factors such as perceiving social support or social pressure are known to affect pain tolerance and pain expression (Krahé et al., 2013). Conceivably, such social factors might also be involved in how fear is expressed. However, more research on avoidance behavior in large-group contexts is necessary to cast light on the group-processes that may affect BAT-performance.

The only two studies that treated dental-related fear in a one-session individual setting so far (Haukebø et al., 2008; Vika et al., 2009), both applied the DAS as a common instrument to measure subjective dental fear and reported pre to post changes of 30.19% (Vika et al., 2009) and 27.11%, respectively (Haukebø et al., 2008). In our study, we found a post-reduction of 16.68% in the DAS for LG-OST completers. At 1-year FU, Vika et al. (2009) reported a fear-reduction of 25.15% and Haukebø et al. (2008) a decrease of 37.35%. In our LG-OST completers, the reduction was 19.08% from pre to FU. Hence, the long-term effect on reduction of subjective fear for the LG- OST is approximately 50–75% of that reported for OSTs delivered in an individual setting.

Other important references for our LG-OST protocol are studies dealing with multi-session (small-) group treatments of dental fear as conducted by Ning and Liddell (1991) and Liddell et al. (1994), Moore and Brødsgaard (1994), and Moore et al. (1996, 2002). However, it is difficult to directly compare their results to our findings: In fact, the Moore et al. studies report DAS-scores as a measure of pre-treatment subjective dental fear, but they only used it as an inclusion criterion and for pre-treatment sample comparability and do not provide information on DAS-score change. Instead, they report change-scores in outcome measures that have not been used in the present study. The Liddell-group only provides visual illustrations, but no numerical parameters, of group-treatment effects on subjective dental fear (DAS). The illustrations provided by Liddell et al. (1994) depict substantial decreases of apparently 35% in the DAS from pre- to posttreatment, which were sustained at follow-up. Although these methodological differences hamper direct comparison, it is reasonable to conclude that the treatment effects of the present study are somewhat smaller than the results reported by the former studies.

In sum, our results corroborate to the suggestion that an LG-OST protocol might sufficiently address the needs for fear treatment of some but not all participants. Therefore, due to its high efficiency, lower costs and threshold of access compared to individual treatment, the LG-OST protocol might be useful as an intermediate step within a future framework of stepped care for phobic fears. So far, there have been some promising efforts to implement stepped care in the field of anxiety disorders, for example, in the treatment of panic or generalized anxiety (GAS); it has been proven that for some patients minimal CBT interventions can be as effective as standard interventions (see Bower and Gilbody, 2005 for an overview). However, besides the benefits addressed, a stepped care approach involves the danger of discouraging those who did not respond to one treatment to proceed to another because they are likely to lose confidence in therapy. In accordance with Newman (2000), we believe that one way to prevent patients’ loss of confidence would be to identify the predictors of treatment response. This, rather than a scattergun approach, might lead to differential treatment recommendations containing stepped care only for those who are most likely to benefit from this approach. For the present LG-OST intervention in dental fear, we identified the level of state fear to be a response predictor. Wannemüller et al. (2016) reported age and the level of pretreatment spider fear to predict treatment response. Interestingly, measures of global clinical impairment such as trait-anxiety or depressiveness or stress did not predict the outcome. However, we encourage more research on LG-OST to optimize the protocol for individual treatment needs.

Our study exhibits several limitations. As customary for Phase I trials, we did not include an untreated or placebo-treatment control group in this feasibility-trial, which involves the danger of confounding post treatment effects with effects of repeated measurement or a regression toward the mean. Furthermore, the number of LG-OST completers who returned for FU-assessment (55.8% of total), was relatively small. As mentioned, we attempted to minimize participants’ doubts to participate in a large group training by guaranteeing anonymity. Therefore, in turn, participants were required to actively waive anonymity to be contacted and scheduled for a follow up appointment. However, we presented data suggesting that dropout was random or non-systematic and that the reported FU-effects can be considered suitable indicators of long-term effects of LG-OST. Finally, we did not measure subjective distress during the BAT, which prevented the depiction of subjective changes in fear perception during the behavioral assessment.

Due to the very high efficiency and general effectiveness of our large-group treatment combining coping with exposure, it is worthwhile to think about how to maximize the potential benefits. We applied a breathing technique as the sole coping strategy, thereby only focusing on bodily fear responses. However, it might have been useful to offer some positive self-verbalizations such as used by De Jongh et al. (1995a) to cover cognitive fear responses and dysfunctional thoughts as well, especially since we observed that the impact of our intervention on dysfunctional cognitions and perceived control in terms of clinical significant change was comparatively small. Furthermore, the content of our exposure material was consistent throughout the trial. Both the video as well as the in sensu exposure dealt with a typical dental surgery, namely tooth filling. Although in many cases this might have been sufficient to trigger dental fear symptoms, a future large-group trial could include diverse dental-related contents such as shorter film-clips. Therefore, a wider range of fear-related situations could be depicted, possibly eliciting fear responses in individuals who did not respond to the video of a standard tooth filling.

## Conclusion

A large-group OST, combining coping with exposure elements proved feasible in highly dental-fearful participants. However, studies investigating the effects of small-group multiple-session treatments or one-session single-treatments on subjective dental reported larger effects than we found in LG-OST.

However, if LG-OST could match the efficacy of highly intensive short treatments delivered in a single setting, for example, by applying a wider array of coping strategies or exposure exercises, LG-OST could be a very useful treatment option due to its exceptional efficiency.

## Ethics Statement

This study was approved by Local Ethics Committee of the psychology faculty of the Ruhr-University Bochum. We used informed consent.

## Author Contributions

Conceived and designed the study: AW, JM, H-PJ, AB, JB, MM, and MV; Analyzed the data: AW; Wrote the paper: AW and SS.

## Conflict of Interest Statement

The authors declare that the research was conducted in the absence of any commercial or financial relationships that could be construed as a potential conflict of interest.

## Footnotes

1 As explained in the Procedure Section, we guaranteed our participants anonymity during LG-OST. Since our LG-OST project was introduced in local media (newspaper, local radio station) it is likely that a few individuals took part in the LG-OST who did not seek treatment in the dental clinic.
